# Neoadjuvant chemotherapy-induced immune remodeling in ovarian cancer: implications for TIL dynamics and combination immunotherapy

**DOI:** 10.3389/fimmu.2026.1757366

**Published:** 2026-02-19

**Authors:** Wanting Zhu, Jiajia Li, Yihua Sun, Qianhan Lin, Yating Sun, Mengyang Xue, Chang Zheng, Xiuling Zhi, Liangqing Yao, Mo Chen

**Affiliations:** 1 Department of Gynecologic Oncology, Obstetrics and Gynecology Hospital of Fudan University, Shanghai Key Lab of Reproduction and Development, Shanghai Key Lab of Female Reproductive Endocrine Related Diseases, Shanghai, China; 2 Department of Pathology, Obstetrics and Gynecology Hospital of Fudan University, Shanghai, China; 3 Department of Obstetrics and Gynecology, Beijing Anzhen Hospital, Capital Medical University, Beijing, China; 4 Department of Physiology and Pathophysiology, School of Basic Medical Sciences, Fudan University, Shanghai, China

**Keywords:** biomarkers, immunotherapy, neoadjuvant chemotherapy, ovarian cancer, tumor immune microenvironment, tumor-infiltrating lymphocytes

## Abstract

Ovarian cancer (OC), particularly high-grade serous ovarian cancer (HGSOC), is among the most lethal gynecologic malignancies, with its therapeutic challenges primarily stemming from a distinctly immunosuppressive tumor immune microenvironment (TIME). Neoadjuvant chemotherapy (NACT) has emerged as a crucial treatment strategy for advanced ovarian cancer; nevertheless, its impact on the tumor microenvironment—especially on tumor-infiltrating lymphocytes (TILs)—is not yet fully understood. As central mediators of antitumor immune responses, the density, composition, and dynamic changes of TILs are strongly associated with chemotherapy response and patient prognosis. Notably, spatial omics studies further revealed that, after NACT, a subset of CD8+ T cells can be confined within “myelonets” microdomains organized by myeloid cells, where interactions such as NECTIN2–TIGIT impose spatial restriction and induce functional exhaustion of T cells, thereby compromising their effective tumor killing. This review aims to systematically summarize the baseline characteristics and heterogeneity of lymphoid- and myeloid-derived TILs in ovarian cancer, elucidate the mechanisms underlying immune remodeling induced by NACT and their complex relationships with clinical outcomes, and further discuss combination therapeutic strategies and biomarker development based on dynamic TIL changes to enhance the clinical application of precision immunotherapy.

## 1 Introduction

Ovarian cancer remains the most deadly malignancy of the female reproductive system, with approximately 75% of patients diagnosed at an advanced stage (FIGO stage III/IV). High-grade serous ovarian cancer (HGSOC) is a highly invasive and heterogeneous subtype, representing over 70% of epithelial ovarian cancers (1). Although the standard treatment approach—comprising cytoreductive surgery combined with platinum- and taxane-based chemotherapy—can induce initial remission in many patients, high recurrence rates and the development of chemotherapy resistance have historically hindered significant improvements in long-term survival, underscoring the critical necessity for novel therapeutic strategies (2). In recent years, research has demonstrated that the distinctive tumor immune microenvironment (TIME) of HGSOC plays a crucial role in its resistance to treatment. Although a certain proportion of tumor-infiltrating lymphocytes (TILs) is present within tumor tissue, their activity is markedly inhibited, resulting in a typical “cold tumor” state (3, 4). Recently, Luo et al. (5) characterized the immune landscape of HGSOC through the application of single-cell and spatial technologies, uncovering extensive immune heterogeneity and an immune evasion mechanism characterized by the enrichment of effector regulatory T cells (eTregs), thereby offering a static snapshot to elucidate its “cold tumor” state. Within this immune landscape, TILs, as key effector cells, possess substantial prognostic and predictive significance in ovarian cancer (6). Numerous studies have confirmed that the density of TILs, particularly CD8+ T cells, as well as the CD3^+^/Treg and CD8^+^/Treg ratios, the development of tertiary lymphoid structures (TLS), and their spatial distribution are strongly correlated with chemotherapy responsiveness and prognosis (7–9). These findings establish the potential of TILs as predictive biomarkers and provide a basis for personalized treatment based on immune profiling.

Neoadjuvant chemotherapy (NACT) provides a distinctive opportunity to investigate the dynamic progression of the tumor immune microenvironment. NACT dynamically modulates TIME through immunogenic cell death, cytokine signaling, and interactions between myeloid and lymphoid cells, converting “cold” tumors into “hot” tumors with active immune responses, thereby creating opportunities for combination immunotherapy (10–12). However, NACT exerts a “dual” influence on the regulation of the immune microenvironment. While stimulating antitumor immunity, it may also trigger immunosuppressive mechanisms, such as upregulation of immune checkpoint molecules like programmed death-ligand 1 (PD-L1) and recruitment of immunosuppressive cell populations such as MDSCs, thereby intensifying immune tolerance. Multiple phase III clinical trials combining chemotherapy with immune checkpoint inhibitors (ICIs) have not demonstrated a substantial enhancement in survival outcomes within the overall population (13, 14). This underscores the limited comprehension of the dynamic progression of the tumor immune microenvironment (TIME) following neoadjuvant chemotherapy (NACT) and the absence of reliable biomarkers capable of accurately predicting treatment efficacy and identifying potential responders.

This review systematically outlines the baseline characteristics and heterogeneity of tumor-infiltrating lymphocytes in ovarian cancer, emphasizing the dynamic remodeling of TILs in aspects of quantity, function, and spatial distribution induced by NACT, and their intricate relationship with chemotherapy response and prognosis. It also explores the potential of combination immunotherapy approaches (such as immune checkpoint inhibitors, adoptive cell therapy, etc.) and the development of biomarkers based on TIL alterations. Establish a novel theoretical framework and translational perspective to enhance the synergistic strategies of chemotherapy and immunotherapy, thereby promoting the advancement of precision immunotherapy for ovarian cancer.

## 2 Composition and regulation of TILs in ovarian cancer

**Figure 1:**
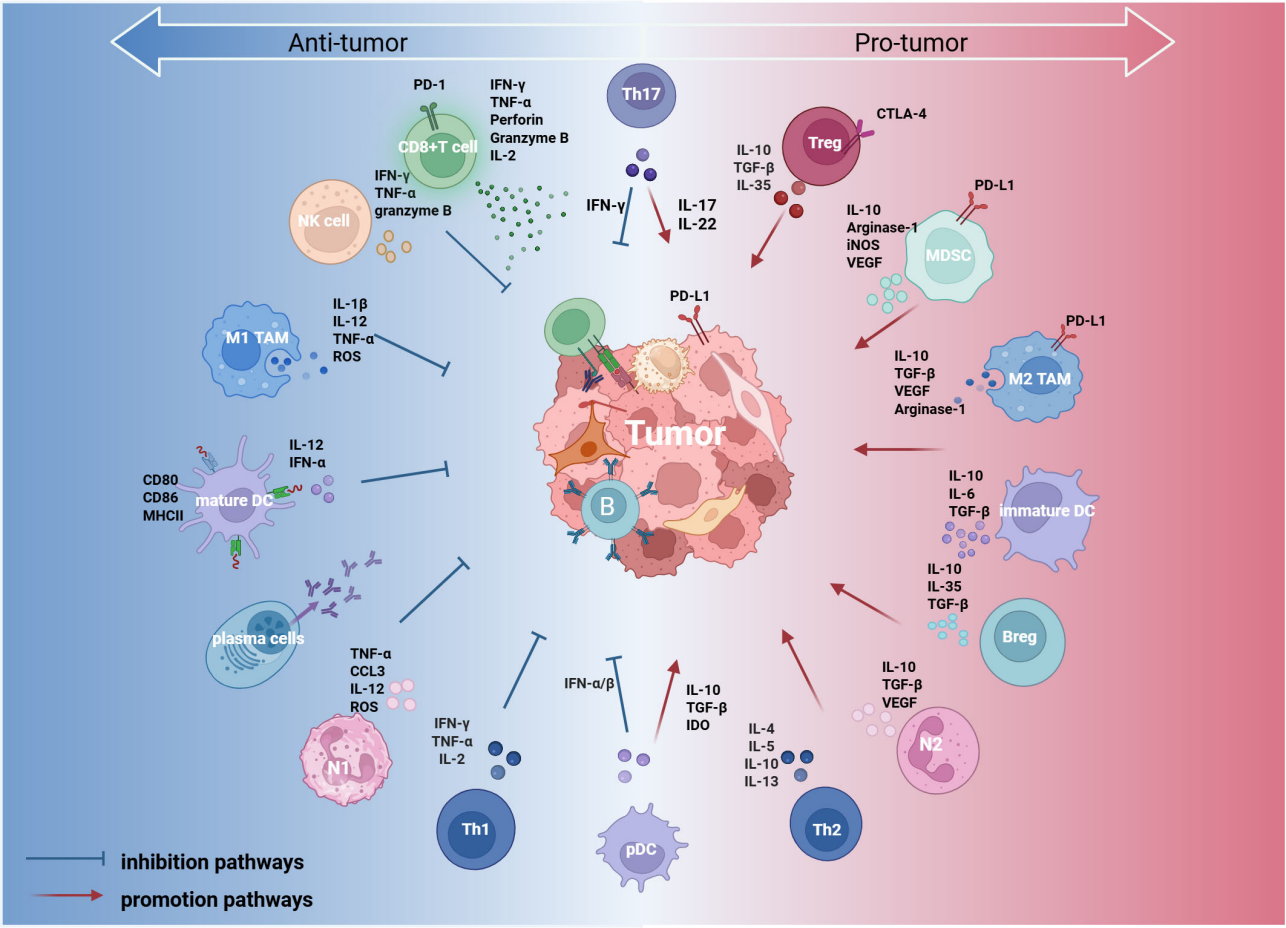
Immune cells within the ovarian cancer tumor immune microenvironment (TIME) are broadly classified into two categories based on their functional roles: pro-tumor (right) and anti-tumoral (left). Certain immune cells, such as Th17 cells and plasmacytoid dendritic cells (pDCs), exhibit dual functions depending on the signals present in the tumor microenvironment (center). These immune cells regulate the tumor immune response through direct interactions with tumor cells and cytokine secretion, thereby influencing tumor progression. This figure was created with BioRender.com. IL, interleukin; TGF-β, transforming growth factor beta; IFN-α/β, interferon alpha/beta; IFN-γ, interferon gamma; MHC, major histocompatibility complex; PD-1, programmed cell death protein 1; PD-L1, programmed cell death ligand 1; CTLA-4, cytotoxic T-lymphocyte-associated protein 4; VEGF, vascular endothelial growth factor; ROS, reactive oxygen species; IDO, indoleamine 2,3-dioxygenase; iNOS, inducible nitric oxide synthase; CCL3, C-C motif chemokine ligand 3; NK, natural killer cell; TAM, tumor-associated macrophage; pDC, plasmacytoid dendritic cell; Treg, regulatory T cell; MDSC, myeloid-derived suppressor cell; Breg, regulatory B cell; Th1, T helper 1 cell; Th2, T helper 2 cell; Th17, T helper 17 cell; N1, N1 neutrophil; N2, N2 neutrophil.

Tumor-infiltrating lymphocytes (TILs) represent a key element of the immune microenvironment in ovarian cancer. Their density, composition, and functional status directly influence the magnitude of the host antitumor immune response and are closely associated with patient prognosis. Based on developmental lineage and immune function, TILs can be broadly classified into lymphoid and myeloid lineages. These cellular populations communicate via intricate crosstalk and signaling networks to create a microenvironmental landscape in which immune suppression and activation coexist (**Figure 1**). To clearly summarize the functional characteristics of these key immune cell subtypes, we compiled the major TIL subtypes and their core functions and clinical significance in ovarian cancer (**Table 1**).

**Table 1:** Major functions and prognostic implications of key TIL subsets in ovarian cancer.

TIL subset	Function	Prognostic significance
CD8+ cytotoxic T cells (CTLs)	Directly lyse tumor cells and constitute a central effector arm of antitumor adaptive immunity.	Higher intratumoral (particularly intraepithelial) density is associated with longer progression-free survival (PFS) and overall survival (OS).
Tissue-resident memory T cells(TRMs)	Persist within tissues and provide sustained local immune surveillance.	Clonal enrichment/expansion is linked to stronger antitumor activity and improved clinical outcomes.
CD4+ T cells	Th1 cells secrete IFN-γ and IL-2 to promote cellular immunity; Th2 cells secrete IL-4/IL-13, suppressing Th1 responses and polarizing TAMs toward an M2 phenotype; T follicular helper (Tfh) cells drive B-cell activation and tertiary lymphoid structure (TLS) formation via IL-21.	Th1 responses generally correlate with more effective antitumor immunity; Th2 is associated with poorer outcomes; higher TLS density is associated with prolonged PFS/OS.
Regulatory T cells (Tregs)	Suppress effector T-cell responses through contact-dependent mechanisms (e.g., CTLA-4) and inhibitory cytokines (e.g., TGF-β, IL-10).	Increased Treg infiltration is commonly associated with tumor progression and worse survival.
B cells	Memory B cells and plasma cells can support antitumor immunity via antibody-mediated effector functions and antigen presentation; Bregs suppress T cells, dendritic cells, and NK cells through IL-10, TGF-β, and high PD-L1 expression, and can promote Treg expansion.	Higher B-cell infiltration and TLS formation are generally associated with a favorable prognosis, whereas Bregs are linked to immune evasion.
Natural killer (NK) cells	Mediate tumor-cell killing without prior sensitization.	NK-cell infiltration correlates with improved survival prognosis, but its function is limited in immunosuppressive tumor microenvironments.
Tumor-associated macrophages (TAMs)	M1-like macrophages are pro-inflammatory and support antitumor immunity; M2-like macrophages mediate immunosuppression by secreting TGF-β, VEGF, and other factors.	M1-like TAMs correlate with better survival, whereas M2-like TAMs are associated with tumor progression and adverse prognosis.
Myeloid-derived suppressor cells (MDSCs)	Suppress CD8+ T- cell and NK-cell function by secreting IL-10 and arginase-1 (Arg-1), disrupt TCR signaling and shape an immunosuppressive environment.	Accumulation of MDSCs is associated with poorer outcomes.
Dendritic cells (DCs)	Professional antigen-presenting cells that initiate and modulate T-cell immune responses.	Prognostic impact depends on maturation/antigen-presenting capacity; impaired maturation can favor tolerogenic programs and attenuate antitumor immunity.
Neutrophils (tumor-associated neutrophils; TANs)	N1-like TANs can enhance immunity (e.g., IFN-γ/TNF-α); N2-like TANs can promote immune evasion and metastasis (e.g., via IL-10/TGF-β).	Tumor-associated neutrophilia is linked to worse prognosis; an elevated peripheral neutrophil-to-lymphocyte ratio (NLR) is an independent predictor of unfavorable outcomes.

### 2.1 Lymphoid-derived TILs

T lymphocytes constitute the primary effector population in antitumor adaptive immunity, with various subsets performing contrasting functions in the regulation of tumor immune responses. CD8+ cytotoxic T lymphocytes (CTLs) are essential effector cells that directly facilitate the destruction of tumor cells. In HGSOC, the density of intraepithelial CD8+ T-cell infiltration is independently correlated with extended progression-free survival (PFS) and overall survival (OS) (15). In recent years, tissue-resident memory T cells (TRMs) have garnered heightened interest owing to their function in sustained immune surveillance. TRMs adhere to the tumor epithelium via the expression of CD103 (αE/β7 integrin), while a subset possessing stem-like characteristics (TRMstem) is capable of self-renewal and sustained replenishment of the effector T-cell population. Their clonal enrichment is strongly associated with increased antitumor efficacy and better prognosis (16, 17). Notably, neoadjuvant chemotherapy (NACT) may reduce the prognostic significance of TRMs by decreasing MHC-I expression on tumor cells (18), indicating that continuous antigenic stimulation is crucial for preserving TRM function and offering new perspectives for combination immunotherapy. CD4+ T cells function as essential regulators of the immune response while exhibiting significant functional heterogeneity. T Helper 1 (Th1) cells produce IFN-γ and IL-2 to activate cytotoxic T lymphocytes (CTLs) and macrophages, and their infiltration is generally correlated with improved antitumor immune responses. Th2 cells facilitate the polarization of M2-phenotype tumor-associated macrophages (TAMs) and inhibit Th1 responses via the secretion of IL-4, IL-5, and IL-13, with elevated Th2 infiltration being strongly associated with reduced progression-free survival (PFS) (19). Follicular helper T cells (Tfh) facilitate B-cell activation and the development of tertiary lymphoid structures (TLS) via IL-21 secretion, thereby serving a vital function in coordinating humoral immune responses (20). In contrast, regulatory T cells (Tregs) serve as essential orchestrators of the immunosuppressive microenvironment. They directly inhibit effector T-cell activity through contact-dependent mechanisms, such as CTLA-4, and through inhibitory cytokines, including TGF-β and IL-10. Elevated Treg infiltration frequently signifies tumor advancement and an unfavorable prognosis (21). Moreover, there is functional heterogeneity among Tregs themselves. CEACAM1+ Tregs are generally highly activated and demonstrate increased immunosuppressive potential, whereas follicular regulatory T cells (Tfrs, CXCR5+) can accumulate within ovarian tumor tissues and inhibit CD8+ T-cell function through IL-10–mediated suppression, thereby further strengthening the immunosuppressive tumor microenvironment (22). Th17 cells play a complex and dual role in ovarian cancer (23, 24): they secrete IL-17 and various other mediators that attract myeloid cells and stimulate angiogenesis, thereby advancing tumor progression; conversely, they can bolster antitumor immune responses by promoting the activation of CD8+ T cells. Overall, increased CD4+ T-cell infiltration is generally associated with a favorable prognosis (25), the overall impact primarily depends on the relative proportions and activation states of specific functional subsets within the tumor microenvironment.

The functions of B lymphocytes demonstrate significant subtype dependence (26): regulatory B cells (Bregs) mediate immunosuppression through the secretion of key inhibitory cytokines, including IL-10 and TGF-β, and by expressing elevated levels of immune checkpoint molecules such as PD-L1, thereby directly suppressing the functional activity of CD4+ and CD8+ T cells, dendritic cells, and natural killer (NK) cells. Meanwhile, Bregs also promote Treg proliferation and facilitate immune evasion (27). Conversely, memory B cells and antibody-secreting plasma cells exert antitumor effects through mediating antibody-dependent cellular cytotoxicity (ADCC) and presenting antigens to stimulate T-cell responses. Research has demonstrated that both tumor antigen–specific and nonspecific IgA responses can effectively suppress ovarian cancer progression by promoting T–B-cell cooperation (28). Notably, B-cell-mediated antitumor immunity spatially relies on the establishment of tertiary lymphoid structures (TLS), which operate similarly to secondary lymphoid organs (SLOs) by orchestrating local adaptive immune responses within the tumor microenvironment. In ovarian cancer, TLS are spatially organized by chemokines such as CXCL13, which recruit B cells, T cells, and follicular dendritic cells (FDCs), thereby facilitating antigen-specific B-cell activation, antibody synthesis, and the enhancement of CD8+ T-cell effector functions. This structural integration of immune cells efficiently coordinates cellular and humoral immune responses, thereby collectively counteracting tumor immune evasion (29). Clinical studies have further demonstrated that, in ovarian and gastric cancers, TLS formation is closely associated with improved patient survival and enhanced sensitivity to immunotherapy, supporting a beneficial immunomodulatory role of TLS across multiple solid tumors (30, 31). However, TLS heterogeneity—including variations in maturation and cellular composition—can substantially influence their immunological efficacy and therefore should be thoroughly considered when assessing their potential as biomarkers for immunotherapy (32).

Although classified within the lymphocyte lineage, natural killer (NK) cells enable rapid innate immune responses, possess the capability to directly identify and eliminate malignant cells without prior sensitization, and serve as a bridge between innate and adaptive immunity (33). Nonetheless, the immunosuppressive tumor microenvironment in ovarian cancer markedly impairs natural killer cell function. Tumor-derived factors such as CA125 impair immunological synapse formation, consequently diminishing natural killer cell activity (34). Furthermore, immunosuppressive cells within the tumor microenvironment, such as regulatory T cells and myeloid-derived suppressor cells, secrete cytokines including IL-10 and TGF-β and upregulate inhibitory receptors such as PD-1 and NKG2A on natural killer cells, collectively resulting in NK-cell exhaustion characterized by decreased proliferation, impaired cytotoxic function, and reduced production of proinflammatory cytokines (35). Tumor-infiltrating NK cells are clinically relevant despite their functional degradation. Research has shown that the co-infiltration of NK cells with CTLs within tumor tissues is associated with enhanced OS and PFS in patients (36). Notably, recent studies have demonstrated that the iron chelator deferiprone (DFP) induces tumor cell DNA damage and activates type I interferon signaling, thereby reversing NK-cell exhaustion, promoting their recruitment and activation at tumor sites, and synergizing with chemotherapy to prolong survival in mice (37). This indicates that reversing NK-cell functional suppression and restoring their cooperation with adaptive immunity may serve as a promising approach to enhance the efficacy of immunotherapy in ovarian cancer.

### 2.2 Myeloid-derived TILs

Tumor-associated macrophages (TAMs) are extremely adaptable immune cells present within the ovarian cancer tumor microenvironment. Their polarization status is strictly regulated by microenvironmental signals. Pro-inflammatory M1-type TAMs secrete cytokines such as IL-12 and TNF-α in response to signals including IFN-γ and lipopolysaccharide (LPS), thereby activating antitumor immune responses. Their infiltration correlates with improved OS (38). Conversely, M2-type TAMs, stimulated by factors such as IL-4 and IL-10, promote angiogenesis, attract Tregs, and suppress immune responses through the expression of PD-L1 and secretion of VEGF and CCL22, which are strongly associated with tumor progression and unfavorable prognosis (39). M2-polarized TAMs generally predominate in ovarian cancer. This polarization state is sustained by signals secreted by tumor cells and is further amplified by metabolic reprogramming, thereby enhancing their protumor functions and rendering them a critical target for reversing the immunosuppressive microenvironment (40).

Myeloid-derived suppressor cells (MDSCs) constitute a heterogeneous group of immature myeloid cells characterized by their immunosuppressive functions. They suppress CD8+ T-cell and NK-cell function by secreting factors such as IL-10 and arginase-1 (Arg-1), which deplete L-arginine and other nutrients essential for T-cell activation, while concurrently impairing TCR signaling. Their accumulation is closely linked to advanced tumors, high-grade tissues, and poor prognosis (41).

Dendritic cells (DCs) are essential antigen-presenting cells that play a crucial role in initiating and modulating antitumor T-cell immune responses. Based on their functional characteristics, DCs can be categorized into several subsets (42): conventional dendritic cells type 1 (cDC1) activate CD8+ T cells through cross-presentation of tumor antigens, forming the foundation of cellular immunity; cDC2 mainly stimulates CD4+ T cells via MHC-II pathways; plasmacytoid dendritic cells (pDCs) play a dual role, secreting type I interferons to activate immunity and potentially expressing PD-L1 or inducing Treg differentiation to promote immune tolerance. However, tumor-secreted factors such as TGF-β and PGE2 frequently impede the maturation of dendritic cells (DCs), compromising their antigen-presenting capabilities and promoting the expression of immunosuppressive molecules, thus transforming them from immune activators into tolerance inducers (43).

In addition, neutrophils, the most abundant myeloid cells, also exhibit functional duality. N1-type neutrophils enhance immune responses by secreting IFN-γ, tumor necrosis factor-alpha (TNF-α), and other cytokines, whereas N2-type neutrophils promote immune evasion and metastasis through the production of IL-10, TGF-β, and other factors (44). Research indicates that neutrophil infiltration correlates with poor prognosis, and a high neutrophil-to-lymphocyte ratio (NLR) in peripheral blood serves as an independent predictor of adverse outcomes (45). Notably, neutrophils exhibit plasticity across different immune states, and their phenotype can be reshaped by immune interventions, thereby enhancing antitumor immunity and improving responses to immunotherapy (39).

### 2.3 The PD-1/PD-L1 pathway

Beyond the intricate interactions of immune cells within the ovarian cancer immune microenvironment, immune checkpoint pathways serve as essential regulators of the antitumor immune response. Specifically, the PD-1/PD-L1 pathway plays a vital role in mediating T-cell exhaustion and facilitating immune escape. Upon antigen stimulation, activated T cells transiently upregulate PD-1 expression to restrain excessive inflammation. However, under conditions of chronic antigen exposure—such as within the TIME—PD-1 remains continuously engaged by its ligands, predominantly PD-L1 (widely expressed on tumor and immune cells) and PD-L2 (present on antigen-presenting cells). This persistent engagement results in sustained inhibitory signaling that promotes an exhausted T-cell phenotype marked by restricted proliferation, decreased cytokine secretion, and reduced cytotoxic function (46). Clinical studies have also confirmed that high-density infiltration of PD-1+ T cells generally indicates a state of exhaustion and correlates with poor prognosis (47). It is important to recognize that the biological significance of PD-L1 expression is contingent upon the context. Research indicates that PD-L1 expression positively correlates with the density of CD8+ TILs and that PD-L1+ tumor cells are associated with prolonged survival outcomes (48). Studies have revealed that PD-L1 is predominantly enriched in tumor-associated macrophages (TAMs) and exhibits strong spatial colocalization with CD8+ T cells, PD-1+ T cells, and Tregs. Notably, patients with malignancies exhibiting regions that are double-positive for PD-L1+ and CD8+ demonstrate markedly enhanced survival outcomes (49). These findings indicate that PD-L1 expression may represent an ongoing intratumoral immune response rather than solely an immunosuppressive condition. Furthermore, in cohorts of HGSOC, PD-L1 expression derived from immune cells has been significantly associated with improved OS, whereas PD-L1 expression on tumor cells does not demonstrate a clear prognostic significance, highlighting the importance of differentiating the sources of PD-L1 expression in the prognostic assessment of ovarian cancer (50).

## 3 Spatial organization, immune phenotypes, and heterogeneity of TILs

### 3.1 Spatial patterns and immune phenotypes of TILs

The spatial distribution patterns of TILs serve as critical determinants of their immune function and clinical prognosis. According to the consensus recommendations of the International Immuno-Oncology Biomarker Working Group, TILs are classified into intraepithelial TILs (iTILs) and stromal TILs (sTILs). The former refers to mononuclear lymphocytes that directly infiltrate the tumor epithelium or cell nests, whereas the latter describes lymphocytes residing within the invasive tumor margin and dispersed throughout the stromal regions between tumor cell nests (51).

**Figure 2:**
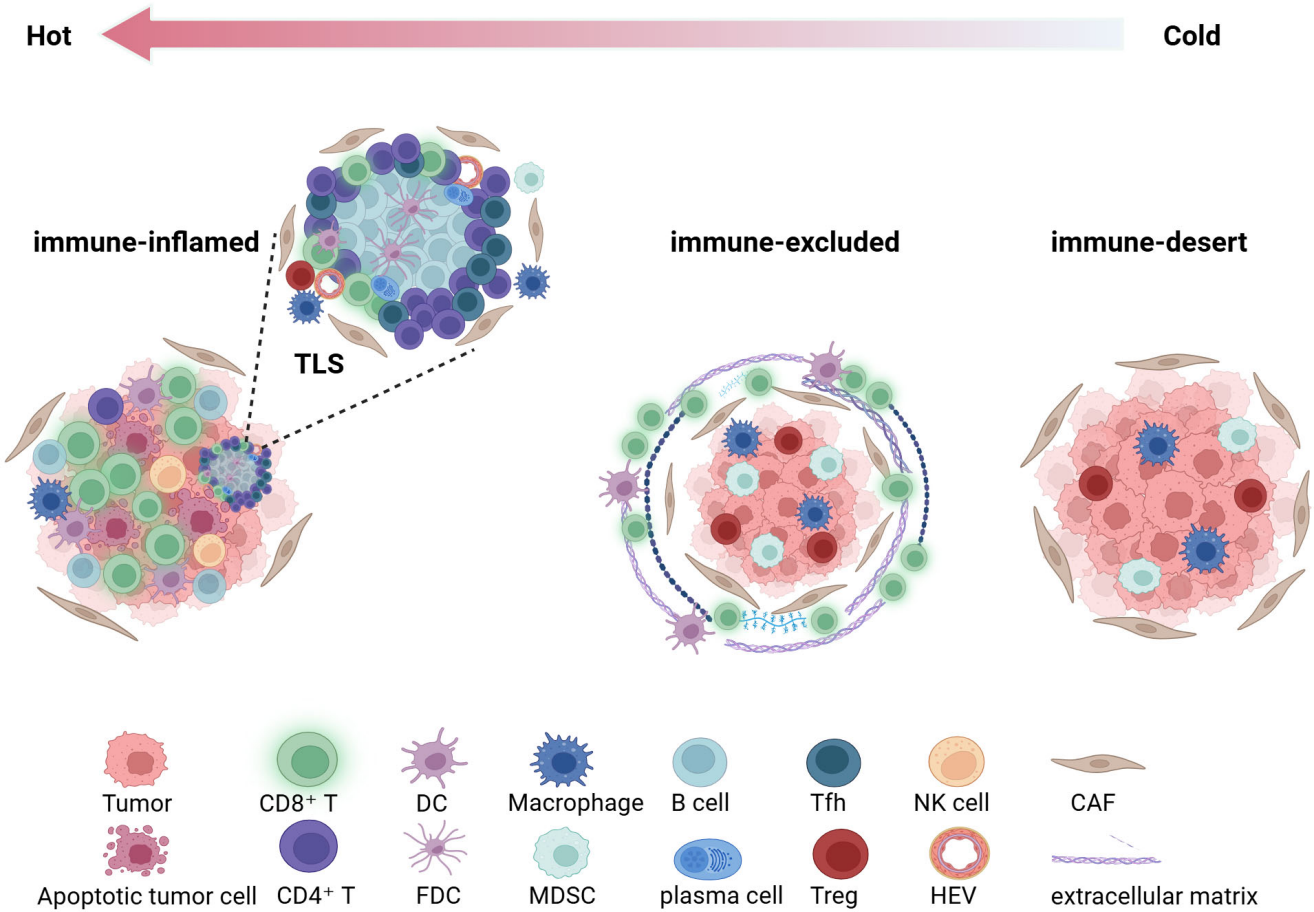
Based on the density and distribution of tumor-infiltrating lymphocytes(TILs), the tumor immune microenvironment is categorized into three immune phenotypes. This figure was created with BioRender.com. TLS, tertiary lymphoid structure; DC, dendritic cell; FDC, follicular dendritic cell; MDSC, myeloid-derived suppressor cell; Treg, regulatory T cell; Tfh, T follicular helper cell; NK, natural killer cell; HEV, high endothelial venule; CAF, cancer-associated fibroblast.

Based on the differences in density and distribution between iTILs and sTILs, TIME is generally categorized into three fundamental immune phenotypes (52, 53) (**Figure 2**): (1) the immune-inflamed phenotype (hot tumor), characterized by a high density of CD8+ iTILs and often accompanied by the formation of TLS, indicating an active antitumor immune response within the tumor, which is generally associated with a favorable prognosis and higher sensitivity to immunotherapy; (2) the immune-excluded phenotype, defined by a high density of sTILs but a marked paucity of iTILs, in which effector T cells are predominantly confined to the tumor stroma and unable to effectively interact with tumor cells. This phenotype implies the existence of physical or functional barriers that facilitate immune evasion; (3) the immune-desert phenotype (cold tumor), characterized by the paucity of T lymphocyte infiltration in both the stromal and epithelial regions, reflecting impaired systemic immune recognition or deficiencies in immune cell recruitment. This phenotype is frequently observed in molecular subtypes such as BRCA wild-type tumors and is commonly associated with poor prognosis and limited responsiveness to immunotherapy.

On this basis, Ghisoni et al. (54) further refined the categorization of the TIME and introduced a novel immune phenotype designated as the “mixed type,” which represents a complex state exhibiting both inflamed and immune-excluded features. This phenotype illustrates the spatial heterogeneity and dynamic equilibrium of the TIME, providing valuable insights into its complex architecture.

### 3.2 Spatial heterogeneity of TILs between primary and metastatic sites

Tumor-infiltrating lymphocytes (TILs) demonstrate pronounced heterogeneity between primary and metastatic lesions in OC. The primary lesions typically present an “immune-cold” phenotype characterized by limited TIL infiltration, predominantly composed of dysfunctional CD8+ T cells. The composition and function of tumor-infiltrating lymphocytes (TILs) are determined not only by the spatial organization of the tumor but also by the patient’s systemic immune status and molecular characteristics. With advancing age, immunosenescence results in diminished T-cell receptor (TCR) diversity, accompanied by persistent low-grade chronic inflammation that fosters an immunosuppressive milieu and facilitates the emergence of an “immune-cold” phenotype (55). Furthermore, systemic inflammatory markers, such as an elevated neutrophil-to-lymphocyte ratio (NLR) in peripheral blood, reflect an imbalance between pro-inflammatory and anti-inflammatory immunity responses. Multiple studies have shown that an elevated NLR is closely associated with adverse clinical outcomes and poor treatment responses, suggesting that systemic inflammation may compromise antitumor immune responses (56, 57). At the molecular level, chronic inflammation triggers activation of the IL6–JAK–STAT3 and TNF–NF-κB signaling pathways, which upregulate immune checkpoint molecules, recruit Tregs and TAMs, suppress the effector functions of CD8+ T cells, and facilitate immune evasion (58). and regulatory T cells (Tregs). In contrast, metastatic sites such as the omentum and peritoneum demonstrate significantly elevated TIL density, albeit with highly heterogeneous cellular compositions and functional states (59). Recent research indicates that omental metastases are predominantly composed of non-specific, minimally clonally expanded “bystander-like” CD8+ T cells, while peritoneal metastases mainly contain exhausted CD8+ T (TOX+PD-1+) cells and FOXP3+ Tregs (60).

In addition, marked spatial heterogeneity has been observed in the expression of immune checkpoints. Research has demonstrated that PD-L1 expression levels are significantly elevated in metastatic lesions—particularly in the omentum—compared to primary tumors, and this elevation is closely correlated with poor prognosis (61). Conversely, peritoneal metastases exhibit a higher proportion of PD-1+ TILs, which is correlated with improved OS; however, PD-L1+ expression, particularly on tumor cells, indicates an immunosuppressive microenvironment and an unfavorable prognosis (62). Notably, activated CD8+PD-1+TOX- cell subsets have been identified as being enriched within the immune microenvironments of metastatic lesions and ascites, suggesting that metastatic sites may possess higher immunogenicity and activation potential (63). Notably, the persistence of circulating IL-6 can be regulated by a “blood-buffering system” formed by dendritic cell–derived soluble IL-6 receptor (sIL-6R), providing a mechanistic explanation for sustained chronic inflammatory signaling (64).

### 3.3 Impact of interpatient heterogeneity on TILs

Among molecular characteristics, the BRCA1/2 mutation (BRCA1/2mt) status and the associated homologous recombination deficiency (HRD) are key factors influencing the immune microenvironment and therapeutic response in ovarian cancer. Patients with BRCA-mutated OC typically exhibit higher tumor mutational burden (TMB) and neoantigen load, accompanied by increased infiltration of CD3+ and CD8+ TILs, as well as upregulated expression of PD-1 and PD-L1 in tumor-infiltrating immune cells (65). Single-cell spatial proteomics has confirmed enhanced spatial interactions between CD8+ T cells and proliferative epithelial cells in BRCA1/2mt tumors, suggesting relatively active immune surveillance (66). Nevertheless, despite the substantial infiltration of CD8+ TILs, these cells frequently exhibit a PD-1+TOX+ terminally exhausted phenotype, with limited capacity for immune reinvigoration following PD-1 blockade therapy (67). Notably, studies have shown that HRD/BRCA-deficient tumors are enriched for IFN-responsive tumor cells and exhibit MHC-II upregulation accompanied by a lack of co-stimulatory molecules (e.g., CD80/86) and increased expression of multiple co-inhibitory ligands, thereby promoting an immunosuppressive/exhausted niche characterized by effector regulatory T cells (eTregs) and terminally exhausted T cells (terminal Tex). In this context, NACT reduces the proportions of terminal eTregs and Tex; however, residual terminal eTregs positively correlate with cancer antigen 125 (CA125), suggesting persistent constraints on deep clinical benefit. In a BRCA1-deficient model, PARP inhibitor combined with eTreg-targeted depletion outperformed monotherapy, indicating that, compared with relying solely on programmed cell death protein 1 (PD-1) blockade to “reinvigorate” terminally exhausted CD8^+^ T cells, HRD/BRCA-deficient populations are more likely to achieve meaningful benefit from combination therapy by disrupting eTreg-driven immunosuppression (5).

In summary, the overall immune status and particular molecular features jointly influence the composition and function of TILs in OC, thereby partially explaining tumor immune heterogeneity and providing a rationale for the stratified design of post-NACT combination therapeutic strategies.

### 3.4 Potential sex-specific differences in TIL dynamics and NACT responses

Ovarian cancer (OC) is female-specific. Therefore, inter-individual differences in endocrine background—particularly menopause-associated hormonal decline, ovarian functional status, and exogenous hormone exposure—may represent key yet often overlooked variables that shape baseline TIL composition and contribute to heterogeneity in chemotherapy responses. Mechanistically, 17β-estradiol (E2) enhances Foxp3 expression in CD4^+^ T cells and expands the CD4^+^CD25^+^Foxp3^+^ regulatory T-cell compartment in an ERα (ESR1)-dependent manner, thereby favoring immune tolerance (68). Menopause is also considered a critical inflection point in age-related immune changes in women, during which more pronounced remodeling of immune composition and phenotype occurs from perimenopause to postmenopause and is closely linked to declining estrogen levels (69). At the tumor level, steroid hormone receptor status may also serve as a layer of stratification that aligns with immunoregulatory features. In epithelial ovarian cancer (EOC), specific patterns of steroid hormone receptor expression are associated with a more immunosuppressive phenotype, characterized by increased FOXP3+ Treg infiltration and upregulated PD-1/PD-L1 signaling, and are linked to poorer prognosis (70). In addition to endogenous hormonal status, exogenous hormonal interventions such as oral contraceptives can also alter the proportion and phenotype of peripheral-blood Tregs (71). Regarding therapeutic response, endocrine receptor context—particularly tumor progesterone receptor isoform B (PR-B) expression—has been linked to platinum chemosensitivity and survival in HGSOC; higher PR-B levels are associated with platinum-sensitive disease and better outcomes, and experimental data suggest that PR-B augmentation and/or progesterone exposure can increase cisplatin sensitivity by promoting cisplatin-related apoptosis (72). Moreover, combined assessment of estrogen receptor α (ER), progesterone receptor (PR), and androgen receptor (AR)—particularly analyses of their co-expression patterns—can further improve the predictive performance for platinum response and prognosis (73). Collectively, these data support incorporating menopausal status, hormone receptor profiles, and exogenous hormone exposure as potential stratification variables or covariates in NACT-related studies to more accurately interpret TIL dynamics and treatment-effect heterogeneity.

## 4 NACT-induced remodeling of the tumor immune microenvironment

### 4.1 Dual immune-modulating effects of chemotherapy

**Figure 3:**
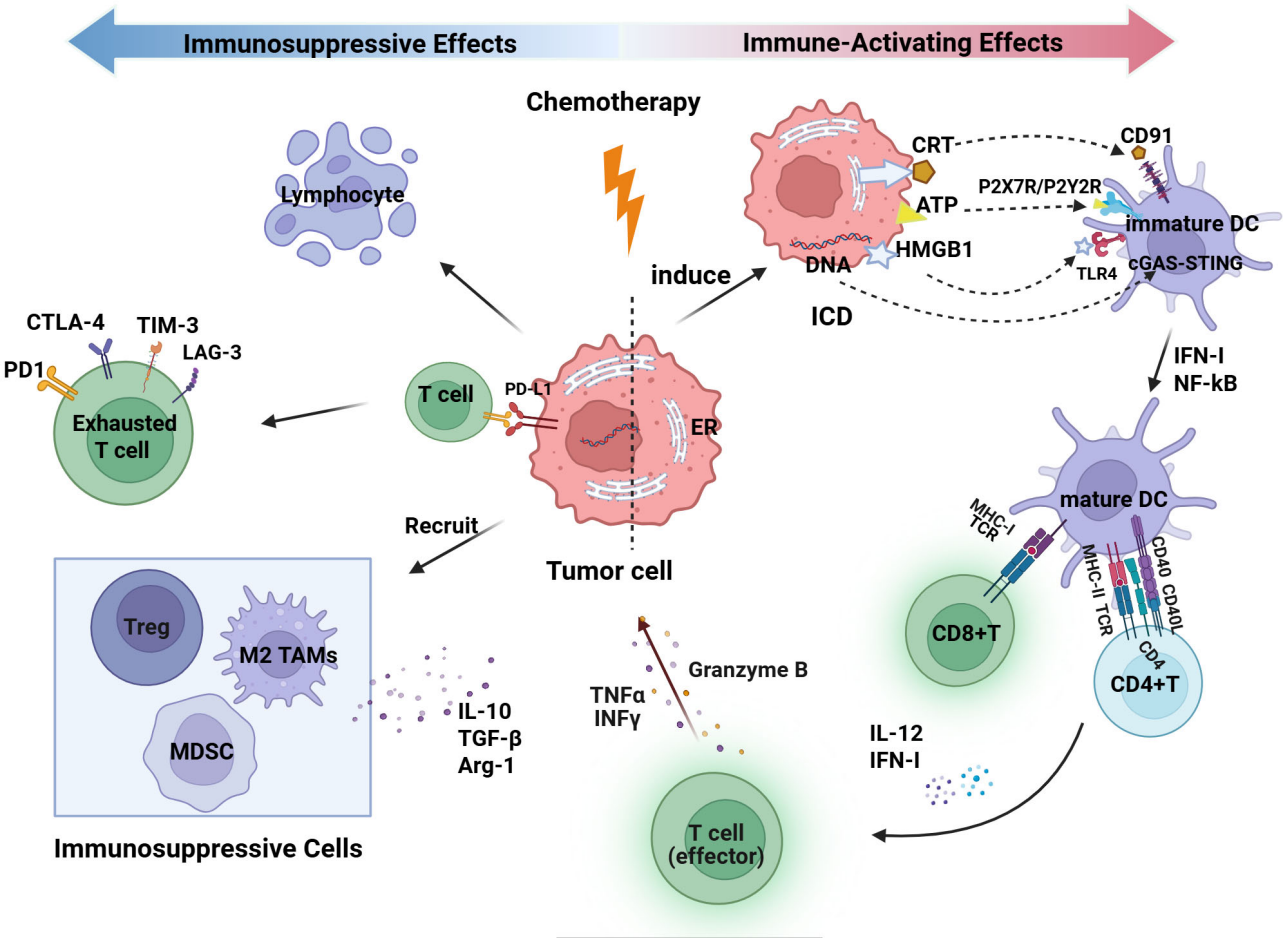
Dual immune modulation effects of Chemotherapy on the ovarian cancer tumor immune microenvironment. This figure was created with BioRender.com. DC, dendritic cell; Treg, regulatory T cell; MDSC, myeloid-derived suppressor cell; TAM, tumor-associated macrophage; MHC, major histocompatibility complex; TCR, T-cell receptor; IFN-γ, interferon gamma; TNFα, tumor necrosis factor alpha; NF-κB, nuclear factor kappa B; IL, interleukin; TGF-β, transforming growth factor beta; CTLA-4, cytotoxic T-lymphocyte–associated protein 4; PD-1, programmed cell death protein 1; PD-L1, programmed cell death ligand 1; Arg-1, arginase 1; TIM-3, T-cell immunoglobulin and mucin domain-containing protein 3; LAG-3, lymphocyte activation gene 3; ICD, immunogenic cell death; ER, endoplasmic reticulum; CRT, calreticulin; ATP, adenosine triphosphate; HMGB1, high-mobility group box 1; P2X7R, purinergic receptor P2X 7; P2Y2R, purinergic receptor P2Y2; TLR4, Toll-like receptor 4; cGAS, cyclic GMP-AMP synthase; STING, stimulator of interferon genes.

Platinum-based chemotherapy combined with paclitaxel is the first-line standard protocol for advanced ovarian cancer. Its effects are not only manifested through direct cytotoxicity but also demonstrate a dual role in reshaping the tumor immune microenvironment: it activates antitumor immunity while also potentially inducing immune suppression (**Figure 3**).

On one hand, chemotherapy induces antitumor immunity by inducing immunogenic cell death (ICD). During this process, certain DNA-damaging drugs (e.g., oxaliplatin) trigger endoplasmic reticulum (ER) stress, leading to the translocation of calreticulin (CRT) to the cell surface, where it serves as an “eat-me signal” recognized by the CD91 receptor on DCs, thereby promoting phagocytosis of dying tumor cells, antigen uptake, and cross-presentation (74). Subsequently, ICD is accompanied by the release of various damage-associated molecular patterns (DAMPs), including adenosine triphosphate (ATP) and high-mobility group box 1 (HMGB1). Extracellular ATP functions as a “find-me signal” by activating P2Y2 and P2X7 receptors, thereby recruiting antigen-presenting cells and promoting the assembly of the NLRP3 inflammasome. HMGB1 interacts with the surface TLR4 and RAGE receptors on DCs, activating the NF-κB and MAPK signaling pathways, thereby promoting DC maturation, increasing the expression of MHC-I/II and co-stimulatory molecules, and augmenting antigen presentation along with the secretion of pro-inflammatory cytokines (75, 76). Additionally, DNA released from tumor cells by chemotherapy can be recognized by the pattern recognition receptor cyclic GMP-AMP synthase (cGAS) in the cytoplasm of dendritic cells. This activates the stimulator of interferon genes (STING) pathway, resulting in the production of IFN-I and enhancing the antitumor immune response (77). Alongside enhancing antigen presentation, chemotherapy additionally modulates immune cell phenotypes. Paclitaxel, through a TLR4-dependent mechanism, induces polarization of tumor-associated macrophages (TAMs) from an immunosuppressive M2 phenotype to a pro-inflammatory M1 phenotype, accompanied by increased expression of TNF-α, IL-6, and nitric oxide (NO), thereby enhancing antitumor immune responses (78). Furthermore, NACT and its combination with immunotherapy have the potential to augment T-cell infiltration and improve functional status, shifting the ovarian cancer immune phenotype from “cold” to “hot,” thus improving the efficacy of immunotherapy (7).

On the other hand, chemotherapy can also induce immune suppression. In addition to the direct effect of bone marrow suppression leading to lymphocytopenia, chemotherapy-induced stress activates inflammatory signaling pathways such as NF-κB and STAT3, which upregulate immune checkpoint molecules like PD-L1 on residual tumor cells and promote T-cell exhaustion (79). More importantly, tissue damage and necrotic responses induced by chemotherapy promote the accumulation of immunosuppressive cells such as MDSCs, TAMs, and Tregs. These cells reshape the immunosuppressive microenvironment by secreting cytokines such as IL-10 and TGF-β, and expressing arginase-1 (Arg-1), thereby limiting the effector function of CD8+ T cells (80).

### 4.2 NACT-induced alterations in TILs and their clinical implications

The regulation of the ovarian cancer immune microenvironment by NACT exhibits complex dynamic characteristics, particularly in terms of the quantity, composition, and functional state of tumor-infiltrating lymphocytes (TILs). These changes in immune cells are closely associated with the patient’s treatment response, pathological remission, and long-term survival outcomes (**Table 2**).

**Table 2:** Changes and prognostic significance of TILs after NACT in ovarian cancer.

Immune markers	Changes after NACT	Prognostic significance	Detection method	Chemotherapy regimen & cycles	Ref.
CD8+ CD4+ FOXP3+ CD20+ CD163+ DC-LAMP+ HLA I	Increased Increased Decreased Increased Increased Increased No significant change	Higher CD8+ T cells and HLA I expression were associated with better OS; The High BinfTinf cluster correlated with improved PFS and OS post-NACT.	mIF, IHC	Carboplatin + Paclitaxel, 3 cycles	(81)
iCD3+ iCD8+ iCD3+CD8+	Increased Increased Increased	Increased post-NACT stromal CD68+CD163- M1 macrophage, stromal CD8+ T cells, and stromal CD8+ PD-1+ cells were associated with benefit from NACT.	mIF	Docetaxel or Paclitaxel + Carboplatin or Cisplatin, ≥3 cycles	(82)
STAB1 FOXP3 CD8+	Increased Increased Increased	Higher STAB1 and FOXP3 infiltration post-NACT were significantly associated with worse PFS.	IHC, scRNA-seq	Carboplatin + Paclitaxel, 3–6 cycles	(83)
iPD-L1	Not reported	High iPD-L1 expression(≥1%) was significantly associated with a better chemotherapy response (CRS 2/3) and shorter PFS.	IHC	Carboplatin + Paclitaxel, 3–6 cycles	(84)
CD8+ CD20+ CD163+ PD-L1+CD68+ CD4+	Increased Increased Decreased Increased Increased	Post-NACT, high CD20+ B-cell density was associated with prolonged PFS, while high M2 macrophage density was associated with shortened PFS. Increased stromal CD4+ T cells post-NACT predicted longer PFS and OS.	mIF	Platinum-based NACT, 2–4 cycles	(85)
CD8+ CD20+ CD4+FOXP3+ CD56+ bright TAMs	Increased Increased Increased Increased No significant change	Increased CD8+ T, CD20+ B, and CD56+ bright NK cells infiltration predicts a good chemotherapy response (CRS 2/3), while increased Treg cells were associated with a poor response (CRS 1).	mIF	Platinum-based NACT, 2–4 cycles	(86)
IL2RAhi-CCL22+Treg CD8+	Increased Decreased	None	scRNA-seq	Carboplatin + Paclitaxel, 2 cycles	(87)
TILs PD-L1 CD8+ FOXP3+	Increased	Increased TILs following NACT were associated with worse PFS and OS.	IHC, RNA-seq	Carboplatin + Paclitaxel, 3–4 cycles	(88)
CD3+/FOXP3+ CD8+/FOXP3+ CD68+/CD163+	Increased	High CD3+/FOXP3+, CD8+/FOXP3+, and CD68+/CD163+ ratios post-NACT were associated with improved PFS.	IHC	Carboplatin + Paclitaxel, 3–4 cycles	(89)
TILs PD-L1 LAG-3 TIM-3 IDO	Increased Increased Increased No significant change No significant change	None	IHC	Carboplatin + Paclitaxel, ≥3 cycles	(90)
CD8+ PD-L1 Immunotype I (PD-L1+/CD8+)	Increased Increased Increased	Immunotype I (PD-L1+/CD8+) serves as an independent predictor of poor prognosis, specifically associated with significantly worse disease-free survival (DFS).	IHC	Carboplatin + Paclitaxel, ≥3 cycles	(91)
MHC-I PD-1+	Decreased No significant change	Low MHC-I post-NACT was associated with poor prognosis	Flow cytometry, IHC, mIF	Carboplatin + Paclitaxel, 6 cycles	(92)
CD3+ CD4+ CD8+ CD45RO+ FOXP3+ PD-1 PD-L1 CTLA-4	No significant change No significant change No significant change No significant change Decreased Increased Increased Increased	Significant T-cell activation was seen in good responders (CRS3), especially CD4+ T cells generating IFN-γ and a decrease in T regulatory cells (FOXP3+). This was linked to better OS and PFS.	IHC, Flow cytometry, RNA-seq	Platinum-based NACT, 6 cycles	(93)
sTILs PD-L1	Increased Increased	High sTILs following NACT associated with Platinum-Free Interval (PFI) ≥ 6 months	IHC	Carboplatin + Paclitaxel, 4 cycles	(94)

IHC, immunohistochemistry; mIF, multiplex immunofluorescence; CRS, chemotherapy response score; PFS, progression-free survival; OS, overall survival.

Among these, changes in T-cell subsets are especially significant, illustrating the complex relationship between immune activation and immunosuppression. It is broadly recognized that NACT significantly promotes the overall infiltration of T lymphocytes within the tumor, with both sTILs and iTILs subsets showing a significant increase compared to pre-treatment levels (95). Among these, the increase in CD8+ T-cell infiltration is a fundamental characteristic, and the balance between CD8+ T cells and Tregs (such as the CD8+/FOXP3+ ratio) holds greater prognostic significance than individual cell density (89, 96). Research has shown that NACT induces changes in TCR clonality, while concurrently enriching functional subsets such as effector memory T cells, suggesting the activation of neoantigen-specific immune responses (97). Furthermore, after NACT, T cell responses to viral antigens are enhanced, accompanied by increased expression of HLA class II molecules on monocytes, indicating an improvement in systemic immune function (98).

However, the immune responses induced by NACT are sometimes constrained by multiple suppressive mechanisms, with the most notable paradox being that, despite an increase in CD8+ T-cell counts, their function frequently becomes exhausted. On one hand, FOXP3+ Tregs significantly increase post-NACT, with the IL2RAhi-CCL22+ Treg subset self-recruiting via the CCL22-CCR1 axis, and further suppressing CD4+ and CD8+ T-cell activity through the CD274-PDCD1 axis (87); on the other hand, activation of the JAK/STAT signaling pathway induced by NACT promotes the accumulation of Tregs, while the upregulation of PD-L1 expression collectively limits the effector function of T cells (88). Spatial transcriptomics analysis demonstrated that CD8a T cells are localized within “Myelonets” microdomains, which are constituted by M1 macrophages via the NECTIN2-TIGIT interaction. This interaction limits their functionality and hinders effective tumor cytotoxicity (99).

In addition to T cells, NACT also exerts significant regulatory effects on other immune cell populations. Studies have shown that B-cell infiltration (B-TILs) increases after NACT, but their antitumor efficacy is highly dependent on the synergistic interactions with other immune cells (85, 100). Furthermore, increased infiltration of B-TILs and IgA plasma cells collectively predicts a better clinical prognosis (20). Following NACT, there is a discernible upward trend in overall NK-cell infiltration, especially within tumor microenvironments rich in CD8+ T cells and B cells. This change is associated with improved survival prognosis in patients (86, 101). At the myeloid immune level, tumor-associated macrophages (TAMs) represent a crucial target of NACT regulation. Research indicates that after platinum-based chemotherapy, the overall density of TAMs decreases, but the remaining TAMs shift towards an antitumor phenotype, characterized by a reduction in CD163+ (M2-like) macrophages and an upregulation of pro-inflammatory pathways, including IL-6 and IL-8 signaling, along with inflammasome activation (e.g., NLRP3/ASC complex formation). These findings suggest that chemotherapy may induce TAM death (such as pyroptosis) and enhance immune stimulatory functions (102). Furthermore, increased infiltration of CD68+CD163- macrophages (M1-like) in the stroma after NACT is significantly associated with treatment benefit (82), suggesting that the accumulation of M1 macrophages may be part of the antitumor immune response induced by NACT. Single-cell RNA sequencing (scRNA-seq) analysis further reveals that the upregulation of Stabilin-1 (STAB1) expression in macrophages induced by NACT is associated with poorer survival prognosis in patients, highlighting the potential of targeting TAMs to enhance chemotherapy-induced immune responses (83). Notably, Lanickova et al. reported that NACT enhances the immunologic adjuvanticity of metastatic lesions by inducing endoplasmic reticulum (ER) stress and promoting calreticulin (CALR) exposure, thereby driving the recruitment of B cells and CD8+ T cells and activating TLS–associated transcriptional programs to promote the transition from early TLSs (eTLSs) to mature TLSs (mTLSs). This process is accompanied by reinforcement of the Tfh–B cell axis and a reduction in immunosuppressive TAMs. Following mTLS formation, PD-1+ CD8+ T cells are preferentially enriched for an ICI–responsive TCF1+ progenitor-like subset with heightened effector output, suggesting that TLS maturation provides a supportive niche that underpins subsequent responsiveness to ICI therapy (103).

At the immune checkpoint level, studies consistently show that PD-L1 expression is broadly upregulated and positively correlated with the densities of CD8+ and CD4+ TILs, as well as CD68+ macrophages, suggesting that its upregulation may reflect a chemotherapy-induced immune activation state (93). However, elevated PD-L1 expression does not necessarily translate into survival outcomes. Although high intratumoral PD-L1 (iPD-L1) expression in preoperative biopsies correlates with a higher chemotherapy response score (CRS2/3), it is also accompanied by a shorter PFS (84). Immune profiling further reveals that when PD-L1 positivity coexists with CD8+ T-cell infiltration (immune subtype I), patients exhibit significantly shorter disease-free survival (DFS), indicating that PD-L1 continues to exert dominant immunosuppressive effects in the presence of T-cell infiltration (91). At the mechanistic level, after NACT, major histocompatibility complex class I (MHC-I) expression on tumor cells is markedly downregulated, leading to impaired antigen presentation, which may partially explain why upregulation of the PD-1/PD-L1 axis fails to confer durable clinical benefit (92). In addition, paired analyses reveal that after NACT, the expression landscape of multiple immune checkpoint molecules (including PD-L1, LAG-3, and TIM-3) is remodeled, providing a biological rationale for combinatorial blockade of multiple checkpoint pathways following NACT (90).

As shown in **Table 2**, existing studies on the prognostic value of tumor-infiltrating lymphocytes (TILs) after neoadjuvant chemotherapy (NACT) have yielded inconsistent conclusions. This inconsistency does not negate the biological relevance of TILs; rather, it is more likely attributable to methodological heterogeneity across studies. Specifically, different studies have used platforms such as immunohistochemistry (IHC), multiplex immunofluorescence (mIF), scRNA-seq, and spatial omics, and differences in resolution mean that IHC often primarily captures changes in “density,” whereas higher-resolution technologies more readily identify functional states (e.g., activation and exhaustion), leading to divergent biological interpretations of TIL dynamics. Meanwhile, the lack of standardized criteria for TIL assessment (e.g., compartment definitions for sTILs vs iTILs, technical approaches, and thresholds for treating TILs as continuous variables versus dichotomizing into high/low groups) (51), together with inconsistencies in PD-L1 evaluation across tumor proportion score (TPS)/combined positive score (CPS)frameworks, positivity cutoffs, antibody clones/detection platforms, and scoring conventions for tumor cells (TC) versus immune cells (IC), can directly alter risk stratification and prognostic inferences (104). In addition, specimen source and treatment chronology are equally critical: the tumor immune TIME differs between primary tumors and omental/peritoneal metastases, and variation in the number of NACT cycles (ranging from 2 to 6) and sampling time points may capture distinct phases of immune activation or exhaustion. Finally, endpoints (PFS/OS/DFS/PFI), follow-up duration, and statistical models are not uniform across studies, and, in particular, whether major confounders—such as residual tumor burden, CRS score, BRCA/HRD status, and baseline TIME phenotypes—are adequately adjusted for can substantially influence the independent prognostic value attributed to TILs. Collectively, these factors indicate that TILs should be interpreted as dynamic, context-dependent biomarkers rather than static quantitative variables, underscoring the necessity of multidimensional and standardized evaluation frameworks.

## 5 Translational and clinical implications

### 5.1 Clinical trials of chemotherapy combined with immunotherapy

Although chemotherapy can promote antitumor immune responses through mechanisms such as inducing ICD, thereby providing a theoretical basis for combination immunotherapy—particularly with ICIs, current clinical studies have not consistently demonstrated survival benefits in patients with ovarian cancer (**Table 2**).

In newly diagnosed patients with advanced ovarian cancer, the combination of chemotherapy with ICIs has not yet demonstrated a definitive survival benefit in the overall population. Two phase III clinical trials—JAVELIN Ovarian 100 (13) and IMagyn050 (14)—evaluated the efficacy of avelumab or atezolizumab in combination with first-line standard chemotherapy (including bevacizumab). Both failed to meet their primary endpoints of PFS improvement. Only the latter trial observed limited benefit in the PD-L1–positive subgroup, which did not translate into an OS advantage, underscoring the limitations of PD-L1 as a standalone predictive biomarker. In contrast, dual immune checkpoint blockade strategies have produced encouraging signals in several phase II studies. In the TRU-D/KGOG 3046 trial, durvalumab plus tremelimumab combined with standard NACT achieved higher pathological complete response (pCR) and R0 resection rates, with prolonged PFS observed in patients with PD-L1 combined positive score (CPS) ≥ 1 (105). However, in the randomized phase II NeoPembrOV trial, although surgical outcomes were similarly improved, no PFS benefit was observed, suggesting that short-term surgical advantages may not necessarily translate into long-term survival gains (106).

In patients with recurrent ovarian cancer, the efficacy of immunotherapy-based combination treatments also remains limited. In platinum-sensitive patients, the phase III ATALANTE/ov29 trial showed that although atezolizumab combined with chemotherapy and bevacizumab resulted in a numerical improvement in median PFS, it neither met its primary endpoint nor improved overall survival (OS), and no significant benefit was observed in the PD-L1–positive subgroup (107). In the platinum-resistant population, the phase III JAVELIN Ovarian 200 trial similarly failed to improve survival in the overall cohort, but more favorable trends in PFS and OS were noted in the “immune-active” subgroup, characterized by PD-L1 positivity and high levels of infiltrating CD8+ T cells, suggesting that therapeutic efficacy may depend on a pre-existing immune-activated state (108). By contrast, several phase II studies have demonstrated the potential value of pembrolizumab-based combination regimens. Combinations with bevacizumab plus cyclophosphamide (109), pegylated liposomal doxorubicin (PLD) (110), or low-dose carboplatin (111) have all achieved notable disease control rates and, in some patients, induced durable responses accompanied by immunologic evidence such as peripheral T-cell reactivation. These findings suggest that optimizing drug combinations and stratifying patients according to immune features may further enhance the therapeutic potential of immunotherapy in recurrent ovarian cancer. Additionally, local immune-delivery strategies—such as intraperitoneal nivolumab combined with HIPEC (112)—and immune gene therapies—such as the IL-12–based therapy IMNN-001 (113)—have demonstrated favorable safety profiles and evidence of local immune activation in early studies, although their true clinical benefits and optimal patient populations require further validation.

Although chemotherapy combined with immune checkpoint inhibitors (ICIs) has shown immune activation and limited clinical benefit in some studies, most phase III clinical trials have yet to demonstrate a survival advantage in the overall population (**Table 3**). This “translational failure” is not solely attributable to upregulation of the PD-1/PD-L1 axis or the limited predictive performance of PD-L1, but more likely reflects the intrinsic complexity of the OC TIME and its dynamic evolution during therapy. OC often exhibits an immune “cold/excluded” phenotype, characterized by insufficient effector T-cell infiltration or spatial restriction, together with enrichment of immunosuppressive cells such as Tregs, MDSCs, and M2-like TAMs, thereby establishing an unfavorable baseline for ICIs responsiveness. NACT can induce ICD and increase antigen exposure, promoting dendritic cell–mediated T-cell priming; however, NACT can also cause myelosuppression and lymphopenia and, under therapeutic pressure, activate pathways such as JAK/STAT and NF-κB, leading to upregulation of PD-L1 and multiple co-inhibitory molecules (e.g., TIM-3 and LAG-3) and the development of adaptive immune resistance. Even when CD8^+^ T-cell numbers increase after NACT, their function may remain constrained by the upregulation of exhaustion-associated molecules, while “myelonets” microdomains formed by myeloid cells can impose spatial segregation through interactions such as NECTIN2–TIGIT, further weakening effector activity (83). In addition, post-NACT downregulation of MHC class I molecules and consequent impairment of antigen presentation may help explain why signs of immune activation do not translate into OS benefit (92). More importantly, most current phase III trials rely on a single biomarker, such as PD-L1, for patient selection and lack precise stratification strategies that integrate multidimensional metrics, including CD8+ T-cell density and spatial distribution, HRD status and TMB. On this basis, disease heterogeneity—such as histologic subtype and platinum-sensitive versus platinum-resistant status—together with suboptimal optimization of timing, dosing, and sequencing of combination regimens, further dilutes signals from potentially responsive subgroups within the overall cohort. Therefore, future efforts should deepen mechanistic understanding of immunosuppression, rationally design combination targeted strategies, and optimize the timing and composition of chemotherapy–immunotherapy regimens; moreover, an integrated biomarker framework should be established to prospectively identify patients most likely to benefit, thereby enhancing the clinical efficacy and OS-translational potential of chemo-immunotherapy combinations in ovarian cancer.

**Table 3:** Main clinical trials of chemotherapy combined with immunotherapy in ovarian cancer.

Trial/ref.	Design	Treatment	ORR(%)	mPFS(months)	OS(months)
TRU-D/KGOG 3046 (105)	Phase II, open-label	Chemotherapy (Carboplatin + Paclitaxel) + Durvalumab	86.7	15.8	NE
KGOG 3045 (114)	Phase II, open-label	Durvalumab + Chemotherapy (Topotecan or Paclitaxel) for high PD-L1 Durvalumab + Chemotherapy + Tremelimumab (75 mg q4w) for low PD-L1 Durvalumab + Chemotherapy + Tremelimumab (300 mg single dose) Durvalumab + Chemotherapy (no PD-L1 screening)	27.6	4.2	27.5
ATALANTE/ov29 (115)	Phase III, randomized, double-blind	Chemotherapy (CbD, CbG, CbP) + Bevacizumab + Atezolizumab Chemotherapy + Bevacizumab + Placebo	NA	13.5 vs 11.23 (HR 0.83, 95% CI 0.69–0.99)	35.4 vs 30.6
ANITA (116)	Phase III, randomized, double-blind	Atezolizumab + Carboplatin doublet + maintenance Niraparib Placebo + Carboplatin doublet + maintenance Niraparib	45 vs 43	11.2 vs 10.1 (HR 0.89, 95% CI 0.71–1.10)	NE
JAVELIN Ovarian 200 (108)	Phase III, randomized, open-label	Avelumab + Pegylated liposomal doxorubicin (PLD) PLD Avelumab	13 4 4	3.7 3.5 1.9	15.7 13.1 11.8
IMagyn050 (14)	Phase III, randomized, double-blind	Chemotherapy (Carboplatin + Paclitaxel) + Bevacizumab + Atezolizumab Chemotherapy (Carboplatin + Paclitaxel) + Bevacizumab + Placebo	93 vs 89	19.5 vs 18.4 (HR 0.92; 95% CI 0.79–1.07)	NE
JAVELIN Ovarian 100 (13)	Phase III, randomized, open-label	Chemotherapy + Avelumab followed by Avelumab maintenance Chemotherapy followed by Avelumab maintenance	NA	18.1 vs 16.8	NE
NCT02865811 (110)	Phase II, single-arm, open-label	Pembrolizumab + PLD	26.1	8.1	18.3
NCT02520154 (117)	Phase II, single-arm, open-label	Neoadjuvant Carboplatin + Paclitaxel → interval debulking surgery → adjuvant Carboplatin + weekly Paclitaxel + Pembrolizumab → maintenance Pembrolizumab	NA	14.9	57.4
NeoPembrOV (106)	Phase II, randomized, open-label	Carboplatin + Paclitaxel + Pembrolizumab Carboplatin + Paclitaxel (NACT → IDS → adjuvant chemotherapy ± Bevacizumab)	72 vs 60	19.4 vs 20.8	49.8 vs 35.3
NCT02853318 (109)	Phase II, single-arm, open-label	Pembrolizumab + Bevacizumab + Cyclophosphamide	47.5	10	NA
NCT03029598 (111)	Phase I/II, single-arm, open-label	Pembrolizumab + Low-Dose Carboplatin	10.3	4.63	11.3
OVATION-2 (113)	Phase I/II, randomized, open-label	IMNN-001 (IL-12 gene therapy) + Chemotherapy (Carboplatin + Paclitaxel) Chemotherapy alone	53.4 vs 57.4	14.9 vs 11.9 (HR 0.79; 95% CI 0.51–1.23)	46 vs 33 (HR 0.69; 95% CI 0.40–1.19)
NCT04072263 (118)	Phase I/II, open-label	Chemotherapy (Carboplatin + Paclitaxel) + TIL infusion (without IFN-α) Chemotherapy (Carboplatin + Paclitaxel) + TIL infusion (with IFN-α)	86	10.7	34.7
NCT02107937 (119)	Phase II, open-label, randomized	DCVAC/OvCa + Chemotherapy (Carboplatin + Paclitaxel) DCVAC/OvCa sequential to Chemotherapy (Carboplatin + Paclitaxel) Chemotherapy alone	NA	20.3 NE 21.4	NE
NCT03959761 (112)	Phase I, open-label	Intraperitoneal Nivolumab + CRS + HIPEC	NA	DFS: 11.0 vs 6.6 (Platinum-sensitive) vs (Platinum-resistant)	NE (Platinum-sensitive) 17.4 (Platinum-resistant)

NA, not available; NE, not estimable; CRS, cytoreductive surgery; HIPEC, hyperthermic intraperitoneal chemotherapy; ORR, objective response rate; mPFS, median progression-free survival; OS, overall survival

### 5.2 Strategies for reprogramming the tumor immune microenvironment

Because the efficacy of chemotherapy combined with ICIs is largely restricted by the broadly immunosuppressive microenvironment and insufficient effector cell function in OC, reprogramming the tumor immune microenvironment to convert “cold” tumors into “hot” tumors has emerged as a critical strategy to enhance immunotherapy responsiveness.

In relieving immunosuppression, Tregs represent a central therapeutic target. Anti–CTLA-4 antibodies can selectively deplete intratumoral Tregs, while CCR4 antagonists reduce their recruitment by blocking chemotactic signaling, thereby alleviating local immunosuppression (22). For M2-like TAMs, which are generally associated with poor prognosis, current therapeutic strategies focus on exploiting their plasticity to repolarize them from a pro-tumor M2 phenotype to an antitumoral M1 phenotype. These approaches include blocking the CSF1R and CCR2 pathways to reduce TAM recruitment, inhibiting PI3Kγ to disrupt pro-tumor signaling and promote M1 polarization, enhancing inflammatory activation through TLR/CD40 agonists, and employing nanoparticle-based delivery systems targeting receptors such as CD206 to selectively eliminate M2-like TAMs (40). Notably, NACT induces overexpression of stabilin-1 (STAB1) on macrophages, and high STAB1 levels are associated with enhanced antigen degradation and an immunosuppressive phenotype. Preclinical studies have shown that targeting STAB1 can delay antigen degradation, promote macrophage polarization toward an antigen-presenting phenotype, and synergize with chemotherapy to improve survival in murine HGSOC models (83). Additionally, targeting the recruitment and suppressive functions of myeloid-derived suppressor cells (MDSCs)—such as by blocking the CCR2/CXCR2 chemokine axis or inhibiting the COX-2/PGE2 pathway—can reduce intratumoral MDSC accumulation, alleviate T-cell suppression, and enhance antitumor responses when combined with ICIs (120). A phase Ib study in glioblastoma (GBM) showed that combining the PDE5 inhibitor tadalafil with chemoradiotherapy significantly reduced peripheral MDSCs and enhanced CD8+ T-cell proliferation and activation, providing clinical evidence for overcoming NACT-associated immunosuppression (121).

To enhance immune effector functions, IL-15 agonists, anti-NKG2A antibodies, and chimeric antigen receptor–engineered NK-cell (CAR-NK) therapies are being developed to restore and strengthen the antitumor activity of NK cells (122). Moreover, dendritic cell (DC) vaccines involve ex vivo maturation and antigen loading of DCs before reinfusion into patients, thereby restoring antigen presentation and enhancing tumor-specific T-cell responses (123). Existing studies have shown that sequential administration of autologous DC vaccines (such as DCVAC/OvCa) after first-line chemotherapy is well tolerated, induces robust antitumor T-cell activation, and delays disease progression compared with chemotherapy alone, thereby providing a promising clinical pathway for post-chemotherapy immunomodulatory strategies (119).

Additionally, several multi-target immunomodulatory agents reported in other tumor models suggest potential complementary approaches for remodeling the TIME. For example, the curcumin analog BDMC-A has been shown in laryngeal carcinoma Hep-2 cells (124) and breast cancer MCF-7 cells (125) to promote apoptosis by modulating the phosphoinositide 3-kinase/protein kinase B/tumor protein p53 (PI3K/AKT/p53) pathway and shifting the balance between BCL2-associated X protein (BAX) and B-cell lymphoma 2 (BCL-2), thereby activating caspase cascades; it can also enhance extrinsic apoptotic signaling via upregulation of Fas ligand (FasL) and caspase-8, and suppress invasion and angiogenesis by inhibiting NF-κB pathway and downregulating cyclooxygenase-2 (COX-2), matrix metalloproteinase-9 (MMP-9), and VEGF (126). Similarly, Tephrosia purpurea extracts can induce mitochondrial membrane potential depolarization and increase caspase-3 expression in HepG2 cells (127), suggesting that natural products may indirectly modulate the microenvironment by directly impairing tumor cell survival. Moreover, in glioma models, the glycopeptide immunomodulator T11TS has been reported to activate the CD2-associated calcineurin–nuclear factor of activated T cells (NFAT) axis and to ameliorate tumor-induced impairment of PI3K–AKT signaling in T cells, thereby supporting T-cell survival and functional restoration. Collectively, these findings suggest that enhancing the intrinsic adaptability and survival programs of effector T cells may serve as a complementary strategy for combination immunotherapy (128, 129).

### 5.3 Adoptive cell therapy

When endogenous immune responses are insufficient to clear tumors, adoptive cell therapy (ACT), which involves ex vivo expansion or engineering of effector immune cells for reinfusion, emerges as a promising therapeutic strategy for advanced ovarian cancer. ACT primarily includes three types: tumor-infiltrating lymphocyte (TIL) therapy, T-cell receptor-engineered T cells (TCR-T), and chimeric antigen receptor T cells (CAR-T).

TIL therapy involves isolating T cells from a patient’s tumor tissue, expanding them ex vivo, and reinfusing them into the body. These cells can recognize multiple tumor-associated antigens and have been extensively investigated in solid tumors such as melanoma and lung cancer, although research in ovarian cancer remains at a relatively early stage (130). Despite its potential advantages, the clinical application of TIL therapy remains limited by prolonged ex vivo culture periods, high costs, and the susceptibility of reinfused T cells to functional impairment within the immunosuppressive tumor microenvironment (131). To address these limitations, researchers have proposed several technical optimization strategies. On one hand, combining TIL therapy with ICIs may alleviate post-infusion T-cell exhaustion and enhance antitumor efficacy (132). On the other hand, non-myeloablative lymphodepletion combined with high-dose IL-2 can markedly increase the yield and functional activity of TILs (133). Building on these technological advances, early clinical investigations of TIL therapy in ovarian cancer have begun to show encouraging signals. In patients with recurrent platinum-sensitive ovarian cancer, combining TIL therapy with platinum-based chemotherapy has achieved an objective response rate (ORR) of 86% and a median progression-free survival (PFS) of 10.7 months (118). The synergistic mechanism may be attributed to chemotherapy alleviating systemic immunosuppression, thereby providing favorable conditions for TIL expansion and activation. Currently, multiple clinical trials are evaluating the safety and efficacy of TIL-based combination strategies, which may ultimately offer more transformative immunotherapeutic options for patients with ovarian cancer.

Genetically engineered T-cell therapies involve modifying autologous T cells to confer specific antitumor activity. CAR-T cells can directly recognize cell-surface antigens independent of MHC presentation and have achieved remarkable success in hematologic malignancies. However, in solid tumors such as ovarian cancer, their efficacy is constrained by multiple factors, including antigen heterogeneity and limited target availability, stromal barriers that impede T-cell infiltration, and an immunosuppressive tumor microenvironment (134). Current research focuses on identifying optimal and stably expressed target antigens, such as mesothelin (MSLN), folate receptor-α (FRα), and MUC16. Meanwhile, next-generation CAR-T designs aim to optimize costimulatory domains, incorporate engineering modules that confer resistance to immunosuppression, integrate cytokine-expression systems, and combine CAR-T therapy with ICIs or chemotherapy to enhance T-cell infiltration, functional activity, and *in vivo* persistence (135, 136). Unlike CAR-T cells, TCR-T cells recognize tumor-derived peptide antigens presented by HLA molecules and can target intracellular ovarian cancer antigens such as NY-ESO-1, MAGE-A4, and WT1. However, their application is limited by HLA restriction and antigen heterogeneity, and remains in the early stages of investigation (137).

### 5.4 TILs as biomarkers

The quantity, spatial distribution, and functional state of TILs not only reflect the baseline immune microenvironment of OC but also exhibit high dynamism during neoadjuvant chemotherapy (NACT)–induced immune remodeling, providing critical biological insights for predicting chemotherapy response and long-term clinical outcomes.

#### 5.4.1 Baseline TILs as predictive biomarkers for chemotherapy response

Studies have shown that chemotherapy-sensitive patients often exhibit higher levels of stromal TILs (sTILs), accompanied by upregulation of immune activation genes such as IRF1, CXCL9, and GZMA, suggesting that a more activated baseline immune state predisposes patients to improved chemotherapy responses (138). Further studies have found that patients with CRS 2/3 responses display higher pre-treatment infiltration of CD8+ T cells and CD20+ B cells, whereas poor responders exhibit enrichment of FOXP3+ Tregs, supporting baseline TIME as a reliable predictor of chemotherapy sensitivity (86). In addition, immune spatial phenotypes also hold predictive value; in immune-excluded tumors, CD8+ T cells remain confined to the stroma and fail to directly contact tumor cells, thereby diminishing chemotherapy effectiveness (139). At the molecular level, high pre-chemotherapy inducible PD-L1 (iPD-L1) expression is significantly associated with higher CRS 2/3 responses (95); whereas BRCA-mutated tumors typically exhibit higher tumor mutational burden and increased TIL infiltration, reflecting a more active immune phenotype that is more likely to achieve pathological responses (CRS3) (140). In summary, baseline TIL levels can help identify an “immune-favorable” population more likely to benefit from NACT, while potentially sparing “immune-cold” patients from ineffective treatment.

#### 5.4.2 Post-NACT TIL changes as predictive biomarkers for prognosis

Changes in the abundance of key effector cell populations after NACT serve as an initial indicator for evaluating immune responses. Studies have shown that increased densities of CD8+ T cells and CD20+ B cells during chemotherapy are independently associated with improved long-term survival (85). However, compared with single quantitative metrics, the CD8+/FOXP3+ Treg ratio—which reflects the balance between effector and suppressive immunity—provides superior prognostic value (89). Importantly, increases in cell numbers must be interpreted in the context of functional status: even when CD8+ T cells accumulate substantially after NACT, high expression of exhaustion markers such as PD-1 and TIM-3 still predicts poor long-term outcomes, underscoring the importance of functional status (99). On this basis, changes in spatial architecture further shape the prognostic significance of TILs. After NACT, CD8+ T cells may become confined within “Myelonets,” microdomains formed by myeloid cells through NECTIN2–TIGIT interactions, restricting their access to tumor epithelium and impairing their activity—an arrangement strongly associated with poor prognosis (99). In contrast, favorable spatial remodeling is characterized by the formation or maturation of tertiary lymphoid structures (TLS). NACT can induce TLS neogenesis in metastatic lesions, accompanied by enrichment of TCF1+ CD8+ T cells (103).

Overall, TILs hold substantial potential as predictive biomarkers in ovarian cancer, yet their clinical utility remains constrained by multiple factors. First, major differences in sensitivity and spatial resolution across assay platforms (IHC, mIF, single-cell approaches, and spatial omics) limit direct cross-study comparability. Second, the lack of harmonized TIL assessment standards—including inconsistent definitions of the sTIL/iTIL compartments, counting units, and “high/low infiltration” cutoffs—directly compromises the accuracy of risk stratification (141). Third, the TIME exhibits marked spatial and temporal heterogeneity, and NACT reshapes the immune landscape, such that single-time-point, single-site sampling and static pre–post comparisons often fail to capture critical dynamic inflection points. Fourth, neglecting functional states can lead to an “immune paradox”: even when CD8+ T cell abundance increases after NACT, antitumor activity may remain limited due to upregulated exhaustion programs or sequestration within myeloid-dominated “myelonets” microdomains, thereby preventing increased infiltration from translating into survival benefit. To address these challenges and advance clinical translation, a standardized, multidimensional assessment framework is warranted. At the pathology practice level, priority should be given to the standardized TIL evaluation workflow proposed by the International Immuno-Oncology Biomarkers Working Group (IIOBWG), which uses H&E-based assessment to unify sTIL/iTIL compartmentalization, inclusion/exclusion regions, and reporting formats, thereby improving cross-study and cross-center comparability and reproducibility (51). At the quantitative level, digital pathology and artificial intelligence should be encouraged to reduce inter-observer variability and enable efficient interrogation of complex spatial features (e.g., cellular proximity and TLS maturity). For clinical stratification, models should move beyond TIL density alone by integrating functional markers (e.g., PD-1 and TOX), cellular ratios (e.g., CD8+/FOXP3+), molecular features (e.g., HRD and TMB), and spatial attributes (e.g., immune phenotypes and TLS) to construct more comprehensive predictive frameworks. Longitudinal studies with paired samples are also recommended to delineate key immune dynamics before and after NACT, thereby informing optimal timing and patient selection for combination immunotherapy.

## 6 Discussion

The treatment of ovarian cancer (OC) continues to face significant challenges, primarily due to the heterogeneity of tumor cells and the immune microenvironment, along with immune evasion mechanisms. Although there has been increasing comprehension of the tumor immune microenvironment (TIME) in ovarian cancer in recent years, the changes in the immune microenvironment induced by neoadjuvant chemotherapy (NACT) and its translational value in immunotherapy remain insufficiently explored. Current research mainly investigates the impact of NACT on the quantification, functionality, and spatial distribution of TILs, but the mechanisms underlying TILs in ovarian cancer are intricate and require further elucidation.

The disparities among existing studies primarily stem from methodological and treatment-related factors. Methodologically, the evaluation of TILs lacks standardized criteria, exhibiting notable variations in detection sensitivity and spatial resolution across traditional immunohistochemistry (IHC), multiplex immunofluorescence (mIF), single-cell sequencing, and spatial omics techniques (51). Additionally, the inconsistency in PD-L1 scoring systems and antibody selection further weakens the comparability among studies (142). In terms of treatment, variations in NACT regimens, number of cycles, time points for efficacy assessment, and sampling sites may all influence the results. More importantly, most studies rely on static comparisons of pre- and post-NACT samples, thereby inadequately capturing the dynamic alterations of TIME. Several studies have shown that although NACT can promote the infiltration of effector cells such as CD8+ T cells, the concurrent exhaustion of their function, the presence of immunosuppressive cells, and the upregulation of PD-L1 give rise to an immune paradox characterized by the coexistence of activation and suppression (91, 99). This paradox is also evident in clinical trials, where although chemotherapy combined with immune checkpoint inhibitors (ICIs) has shown immune activation and clinical benefits in some studies, most phase III trials have failed to demonstrate survival advantages in the overall population (13, 14). This indicates that dependence solely on chemotherapy-induced immune augmentation is inadequate to surmount the immune evasion mechanisms in ovarian cancer.

Future research should develop standardized analytical workflows that go beyond conventional static sample comparisons and incorporate high-throughput methods such as single-cell sequencing and spatial transcriptomics to dynamically investigate the spatiotemporal progression of the immune microenvironment and pinpoint critical immune response nodes. Simultaneously, a precise immune stratification system should be established utilizing multi-omics data, and in conjunction with AI-assisted pathological image analysis, a standardized TIL evaluation framework suitable for clinical application should be developed to accurately identify patient populations who will genuinely benefit. Ultimately, treatment strategies should develop into mechanism-based combination regimens, such as integrating neoadjuvant chemotherapy with immune checkpoint inhibitors, adoptive cell therapy (ACT), and targeted immune cell therapies, while optimizing treatment timing and combinations according to dynamic biomarkers to enhance precision immunotherapy in ovarian cancer.
